# Colorectal Cancer: A Systematic Review of the Current Situation and Screening in North and Central Asian Countries

**DOI:** 10.7759/cureus.33424

**Published:** 2023-01-05

**Authors:** Arunima Dutta, Rebecca Pratiti, Atefeh Kalantary, Armen Aboulian, Shant Shekherdimian

**Affiliations:** 1 Department of Internal Medicine, Franciscan Health, Seattle, USA; 2 Department of Internal Medicine, McLaren Health Care, Flint, USA; 3 Department of Surgery, Kaiser Permanente, Woodland Hills, USA; 4 Department of Surgery, Ronald Reagan University of California, Los Angeles (UCLA) Medical Center, Los Angeles, USA

**Keywords:** cancer registries, cancer screening, north and central asian countries, low-middle income countries, colorectal neoplasms

## Abstract

The prevalence of colorectal cancer (CRC) is increasing in the past few decades. A significant proportion of this increase is from low to middle income countries (LMIC). CRC prevalence is also increasing in North and Central Asian Countries (NCAC). Screening for colorectal cancer has decreased CRC mortality but data regarding screening practices in NCAC is limited.

A literature search was conducted in PubMed/Medline, Embase and Cochrane for current colorectal cancer screening practices in NCAC. Incidence and mortality rates were derived from public health agency websites to calculate age-standardized CRC mortality-to-incidence ratios. Web-based online break-point testing defined as statistical major changes in CRC mortality trends was completed.

Among the 677 screened studies, 37 studies met the criteria for inclusion for review. CRC screening in NCAC is not organized, although most countries have cancer registries. The data availability is scarce, and most data is prior to 2017. Most studies are observational. There is minimal data about colonoscopy preparations, adenoma detection and complications rates. The polyp detection rates (PDRs) and adenoma detection rates (ADRs) seem low to optimal in this region. Commonly measured outcomes include participation rate, fecal immunochemical tests (FIT) positivity rate and cost-benefit measures. Lower mortality-to-incidence ratios is seen in countries with screening programs. Kazakhstan and Lithuania with screening programs have achieved breakpoint suggesting major changes in CRC mortality trends.

Data about CRC screening varies widely within NCAC. High human developmental index (HDI) countries like Lithuania and Estonia have higher incidence of CRC and mortality. Seven NCAC have CRC screening programs with most utilizing non-invasive methods for screening. Data collection is regional and not organized. The ADR and PDR are low to optimal in this region and cancer detection rates are comparable to other high-income countries (HIC). CRC detection rate is 0.05% for screening in Kazakhstan and 0.2% for screening in Lithuania. Very limited information is available on the actual cost and logistics of implementing a CRC screening program. All NCAC have a cancer registry, with some having a high-quality registry showing national coverage with good validity and completeness. Establishing guideline-based registries and increasing screening efficacy could improve CRC outcomes in NCAC.

## Introduction and background

Worldwide colorectal cancer burden

In 2020, there are more than 1.9 million new cases of colorectal cancer (CRC) and 935,000 deaths reported worldwide [1]. The highest incidence rates of colon cancer are in European regions, Australia, New Zealand and Northern America. Rectal cancer incidence rates have similar distribution although rates in Eastern Asia rank among the highest [1]. As per recent 2020 data, female breast cancer (11.7%) is the most commonly diagnosed cancer followed by lung (11.4%) and colorectal (10.0%) cancer. Lung cancer is still the leading cause of cancer death (18%) followed by colorectal cancer (9.4%) [1].

Trends in different regions of the world

The number of people diagnosed with colon and rectal cancer doubled from 1990 to 2013, with most of the increase resulting from an aging and growing population. Studies have shown CRC incidence relates to Human Development Index (HDI) and higher CRC burdens are seen in high-income countries (HIC) [2,3]. Incidence rates are rising in many countries in Eastern Europe, southeastern and south-central Asia and South America due to changes in lifestyle factors including diet and physical activity [4-8]. Decline in colorectal cancer incidence in some high-incidence countries has been primarily due to increasing uptake of screening practices and secondarily due to healthier lifestyle choices [9,10]. The favorable trend of decreasing colorectal cancer is being impeded by the recent rise of early-onset colorectal cancer (<40 years) in many high-income countries like the USA, Canada and Australia [11]. This has led to the reforming of screening guidelines and lowering the eligible age for screening from 50 to 45 years as per the American Cancer Society [1].

The North and Central Asian Countries (NCAC) countries, namely Armenia, Azerbaijan, Belarus, Estonia, Georgia, Kazakhstan, Kyrgyzstan, Latvia, Lithuania, Moldova, Russia, Tajikistan, Turkmenistan, Ukraine, and Uzbekistan, fall in different regions - Eastern Europe, Central Asia, Transcaucasia and Baltics. The NCAC countries can be divided depending on the HDI as follows: Very high HDI: Estonia, Lithuania, Latvia, Kazakhstan, Russia, Belarus, Georgia; High HDI: Ukraine, Armenia, Azerbaijan, Moldova, Uzbekistan, Turkmenistan; Medium HDI: Kyrgyzstan, Tajikistan [12]. 

Colorectal cancer screening

CRC screening aims to detect early-stage CRCs and precancerous lesions in asymptomatic people who have no prior history of cancer or precancerous lesion. The five-year survival rate for CRC is dependent upon the stage at diagnosis, varying from 90.1% for Stage 1 (localized) to 13.1% for Stage 4 (metastatic) [13]. CRC can be prevented to a large extent by screening and removing suspicious polyps and masses [14]. In a German study, 180,000 CRC cases were prevented by sustained 10 years of screening colonoscopies [15]. CRC screening tests are ranked in three tiers based on performance features, costs and practical consideration.

The U.S. Multi-Society Task Force on Colorectal Cancer recommends CRC screening to begin at age 50 and discontinue at age 75 or when life expectancy is <10 years [14,16]. The various screening tests can be divided into invasive vs noninvasive [17]. Non-invasive tests are guaiac fecal occult blood test (gFOBT), fecal immunochemical tests (FIT) and FIT-DNA. Dietary factors, type (manufacturer) and threshold for positivity affect the sensitivity and specificity of the tests [18]. FIT has a higher sensitivity and specificity to detect adenomas and CRCs compared to gFOBT [19]. Sensitivity of gFOBT is 62-79% and specificity is 87-96% [19,20], while for FIT, they are about 79% and 94% respectively [21]. Reduction in CRC mortality is 22-62% with FIT compared to 9-22% with gFOBT [17]. Colonoscopy, though an invasive test, is the gold standard for CRC detection with a sensitivity of 88-98% and specificity of 92-99% [17]. In the study by Aronsson all screening strategies showed cost effectiveness as compared to no screening, though colonoscopy was more cost effective than FIT [14].​

Screening strategies can be divided into two major types, namely organized and opportunistic screening [14,16]. Organized screening involves testing at the population level with gFOBT or FIT and following up on those who are positive with further testing like colonoscopy. Though more efficacious, this type of screening requires significant investment, organization and coordination [22]. Hence, it has been implemented mostly in HICs including France, Hungary, Austria, Belgium, Sweden, Poland, England, etc. [16]. On the other hand, opportunistic screening is done on an ad hoc basis either free of cost or fee-for-service [23]. Countries with opportunistic screening include USA, Turkey, Greece, Germany and Switzerland. Colonoscopy is the most common test of choice in this form of screening. In Europe, 16 countries have organized while nine follow opportunistic screening methods [16].

Most HICs have developed guidelines for CRC, and many have associated quality and surveillance oversight with continuous reassessment and modification processes in place [22]. The situation in low- to middle-income countries (LMICs), however, is significantly different. LMICs are having an increase in risk factors for colorectal cancer such as obesity, smoking, and alcohol use, though few LMICs have a CRC screening program in place with optimal implementation and utilization [16,24]. Mechanisms to assess efficacy of such programs and to conduct real-time assessments with continual process improvement are often lacking. As a result, mortality from CRC is still high. Additionally, CRC prevention planning is based on prioritization by disease burden, available resources and infrastructure. Screening by a low-cost non-invasive test like FOBT or FIT seems to be a better option for LMICs [21].

Rationale for literature search

Although the incidence of colorectal cancer is rising in NCAC, no dedicated systematic review is available that discusses the colorectal cancer disease burden and screening methods in this region. The purpose of the study was to evaluate the CRC risk burden in NCAC, compiling the most recent screening practices in the region and assessing barriers to diagnosis and treatment for CRC in NCAC.

## Review

Methodology

Literature Search

For the literature review, with guidance from a medical librarian, we conducted a systematic search from March 2020 to May 2020, with no restrictions on article publication date. We searched PubMed/Medline, Embase and Cochrane Library. Literature search was performed to integrate the information about screening practices in NCAC. The data for current CRC incidence and mortality were derived from Global Cancer Observatory which provides tabulation and graphic visualization of the GLOBOCAN database for 185 countries and 36 cancers (as well as all cancers combined), by age and sex [25]. Further an online breakpoint analysis was performed for NCAC to assess if there are any significant changes to CRC mortality in NCAC in the presence or absence of a screening program. The search strategy included the following terms and medical subject headings: Armenia, Azerbaijan, Belarus, Estonia, Georgia, Kazakhstan, Kyrgyzstan, Latvia, Lithuania, Moldova, Russia, Tajikistan, Turkmenistan, Ukraine, Uzbekistan, Colon Cancer, Rectal Cancer and Colorectal Cancer.

Study inclusion and exclusion criteria: We limited our search strategy to studies with English abstracts. We included (1) randomized controlled trials (RCTs), cohort studies, case-control studies, observational studies, conference abstracts, modelling studies using statistics and studies with pre-post design; (2) studies conducted in at least one NCAC; (3) studies on CRC screening and (4) studies on CRC diagnosis. We excluded (1) Studies that were not related to CRC screening; (2) Non-human studies; (3) Studies about colorectal cancer treatment and (4) Case reports. All data cited confirmed to the Helsinki Declaration on human experiments.

Article selection, data extraction and analysis: Studies were reviewed, and articles were selected based on the selection criteria outlined above. Articles were deemed relevant for inclusion by initially scanning the titles and abstracts. If the criteria were not easily identifiable from the titles and abstract, the article was included, and a full article review was then conducted to confirm eligibility. Eligible studies were examined for study location and relation to CRC screening and prevention. Discrepancies were resolved by discussion.

Online Break-Point Analysis of CRC Mortality Trends in NCAC

The data extracted and reported in the results sections of the article is from the website https://gco.iarc.fr/ [25] and http://dep.iarc.fr/WHOdb/WHOdb.htm [26] as per the data that was available on the websites as of 05/2020. The data is available for different countries in different time periods. For most countries, mortality trends are available from 2010 to 2015. For some countries, data is available up to 2017 while for some no data about CRC mortality trends is available. The trends have been depicted with latest available mortality data. If the countries achieved a breakpoint where a major change in CRC mortality was noticed, a green line had been placed at that point. This data is derived from International Agency for Research on Cancer (IARC) and has limitations as stated in the source website and is for research purposes only [26].

Results

Literature Search

**Figure 1:**
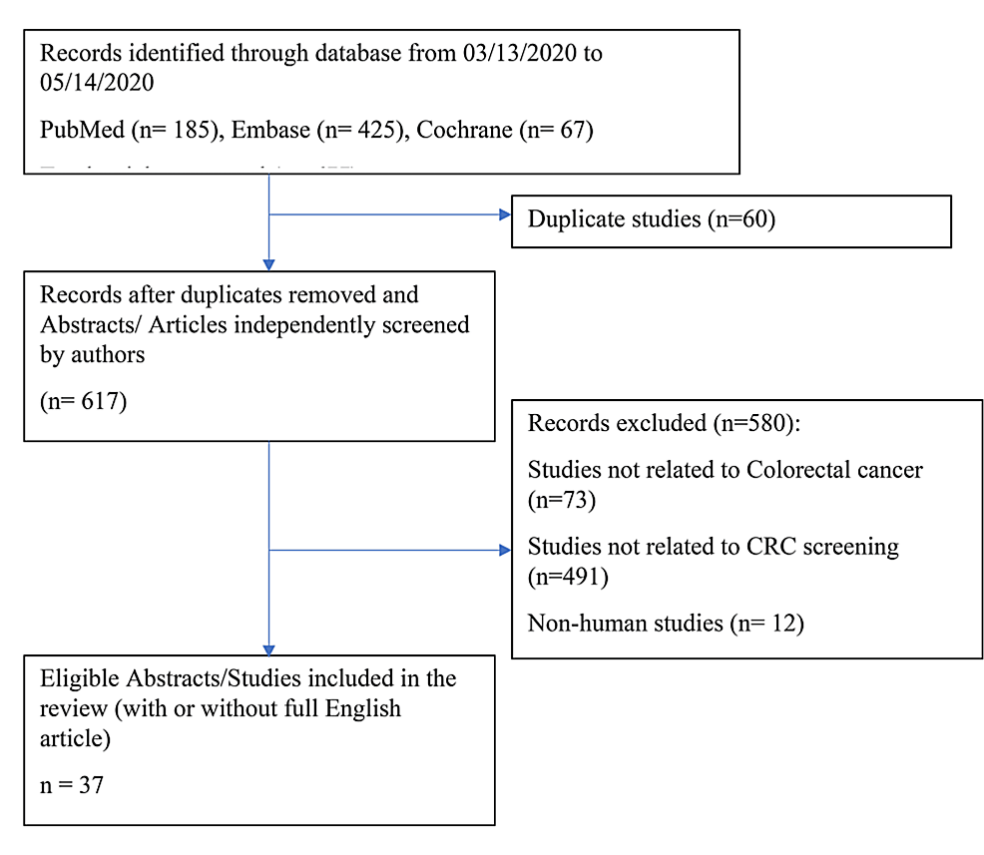
Preferred Reporting Items for Systematic Reviews and Meta-Analyses criteria (PRISMA) flow diagram CRC: colorectal cancer

A total of 677 studies were screened, and 37 studies met the eligibility criteria for inclusion. Because of limited data available on the topic, if the study abstract met inclusion criteria, the abstract was included even though a full article was not available. Two of the included studies were based on statistical models for CRC-related preventive measures. The availability of data about CRC screening and prevention varies widely by country with some countries having no published data. Most studies were observational studies. None of the studies were randomized. Commonly measured outcomes were objective including participation rate, FIT positivity rate and cost-benefit measures. Figure 1 shows the Preferred Reporting Items for Systematic Reviews and Meta-Analyses criteria (PRISMA) flow diagram. Most of the excluded studies were related to diagnosis or treatment modalities for CRC, CRC prevalence, epidemiological studies and studies on genetic markers for familial colon cancer.

Current CRC Incidence and Mortality in NCAC

**Figure 2:**
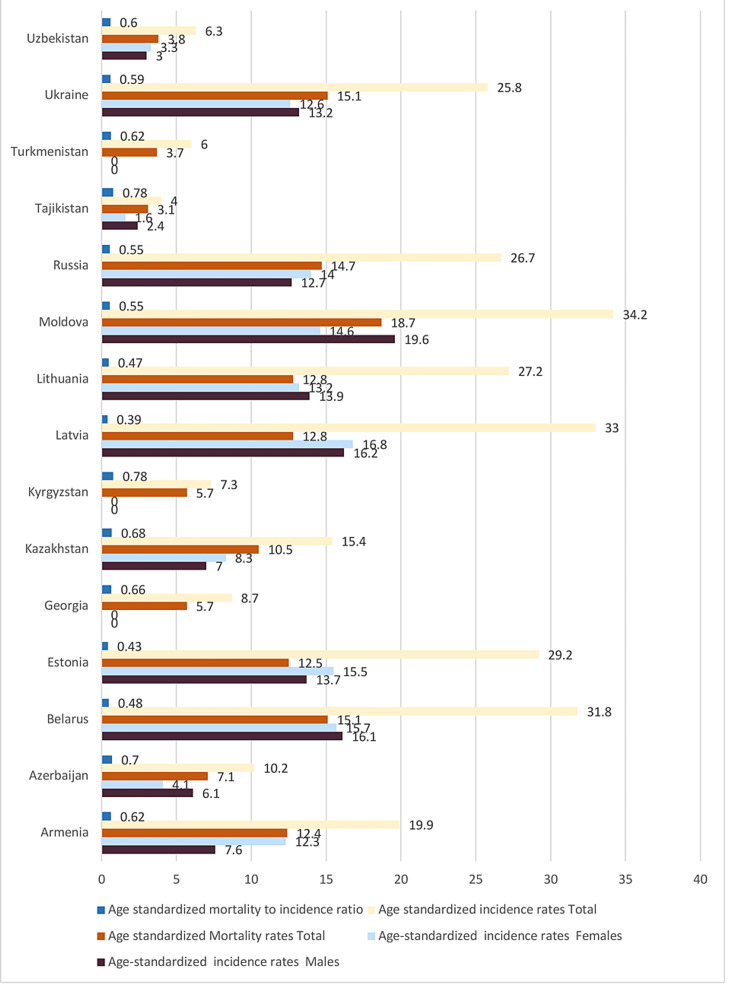
Current CRC incidence and mortality in North and Central Asian countries Data from Global Cancer Observatory, Globacon 2018 [25] CRC: colorectal cancer

Figure 2 provides the data from Global Cancer Observatory, Globacon 2018 [25]. There is a clear trend for lower mortality-to-incidence ratios for countries with a screening program including Estonia, Georgia, Kazakhstan, Lithuania, Latvia, Russia and Ukraine.

Online Break-Point Analysis of CRC Mortality Trends in NCAC

**Figure 3:**
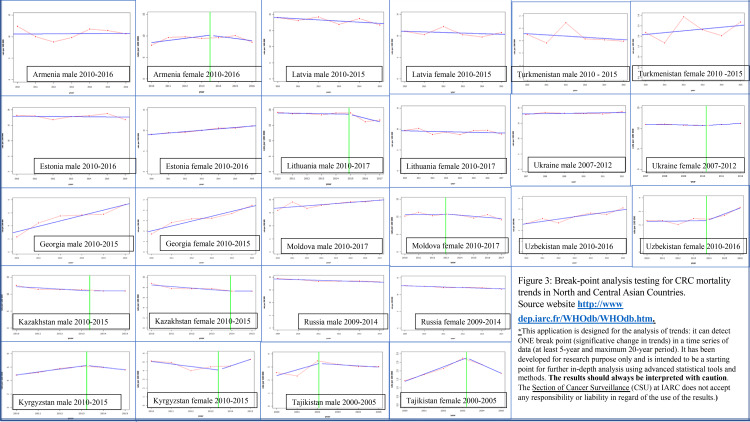
Break-point Analysis Testing for CRC Mortality Trends in North and Central Asian Countries Source website http://www dep.iarc.fr/WHOdb/WHOdb.htm. *This application is designed for the analysis of trends: it can detect ONE break point (significative change in trends) in a time series of data (at least 5-year and maximum 20-year period). It has been developed for research purpose only and is intended to be a starting point for further in-depth analysis using advanced statistical tools and methods. The results should always be interpreted with caution. The Section of Cancer Surveillance (CSU) at IARC does not accept any responsibility or liability in regard of the use of the results.)

‘Breakpoint achievement’ in terms of colorectal cancer mortality means there is a drastic change in the mortality. As shown in Figure 3, in Kazakhstan, both male and female CRC mortality trends have achieved breakpoint indicating statistically significant changes. Kazakhstan has a voluntary nationwide CRC screening program. Lithuania had shown decrease in CRC mortality with breakpoint for males only. Lithuania also has a screening program since 2009. Kyrgyzstan and Tajikistan though do not have a screening program, breakpoint is reached for both males and females for significantly decreased CRC mortality except for Kyrgyzstan females. Further studies are needed to evaluate this trend. Since the mortality to incidence ratio is high for these countries suggesting possible other underlying causes for mortality trend changes. Further, for both these countries, CRC is not one of the most prevalent cancers. Some of these countries with screening program may not have reached breakpoint because of insufficient time since starting screening program to see benefit in mortality trends or there could be possible barriers to CRC screening or treatment. Azerbaijan and Belarus do not have the breakpoint analysis available.

Country-Wise Report on CRC Data and Screening in NCAC

1. Armenia: CRC screening: No form of CRC screening is currently available, though CRC ranks third in cancer incidence with an age standardized incidence rate of 19.9 and mortality-to-incidence ratio of 0.62. Currently a pilot study for CRC screening is being planned to be implemented for evaluating feasibility and cost-effectiveness. 

2. Azerbaijan: In a prospective study of patient undergoing colonoscopy in one medical center, 3.6% of patients were diagnosed with colon cancer. Feasibility of screening for younger population with familial form of colonic polyposis in this region needs further studies [27]. A significant percentage of 57.2% had right sided colon cancer. None of the alarm symptoms including diarrhea, abdominal pain, rectal bleeding, constipation, altering diarrhea and constipation, history of cancer, known irritable bowel disease, history of polyp and fissure or family history of cancer were predictors for cancer or polyps, though the age of the patient and unexplained anemia independently predicted cancer. Since the study was done in one medical center, the results may not be generalizable to normal population [28]. While a recent study after more westernization of diet showed a cancer prevalence of 4.9% amongst patients who underwent colonoscopy for lower gastrointestinal symptoms, though almost 32.7% of colonoscopies were normal [29].

3. Belarus: From recent International Conference on Cancer Nursing data, Belarus has a high-quality population-based cancer registry. Breakpoint analyses for changes in cancer trends are not available for Belarus, though data suggest CRC-related mortality is improving in Belarus [30]. Another study recruiting patients seeking care for intestinal symptoms in a medical center found, among colonoscopies done in age group 45-70 years, 51.8% had polyp. 47.3% of these polyps were adenomatous with potential for neoplastic transformation [31]. Excessive colon cancer risk was found amongst the male liquidators who were mobilized to clean up the most contaminated territory in the Gomel region affected by Chernobyl accident as compared with a corresponding adult population of the non-affected (Vitebsk) region (p<0.05) [32].

4. Estonia: CRC screening in Estonia is not fully implemented. A pilot program was started in 2016 to screen men and women at age 60 with FOBT every two years, followed by colonoscopy in case of positive screen. A recent health policy article had confirmed the implementation of a pilot program for CRC screening [33]. European Union Guidelines for CRC screening are followed. The screening program includes screening by invitation and reminders are sent in two months. Appropriate patient notification of abnormal results, referral, follow up and updating the data is performed every three months [34]. No data is available from this screening program.

5. Georgia: Georgia has CRC screening program since 2006, based in Tbilisi as centralized and regional outside Tbilisi. The breakpoint analysis does not show any major change in the CRC trends. Data from this screening program from 2011 showed, out of 1,368 FOBT conducted, 54 (4%) subjects were positive. All the FOBT-positive subjects were referred to specialized clinic and underwent colonoscopy (100%). Men and women between 50-70 years were screened and apparently the participation rate was 100% with almost 89% screening being conducted in National Screening Center. This model seems to have the benefit of dedicated government funding and trained people with a dedicated screening center for good participation rate, public awareness and efficacy of screening test [35].

6. Kazakhstan: Kazakhstan has a voluntary nationwide FOBT-based CRC screening program since 2011 for people over 50 years to be done every two years. Breakpoint analysis suggests Kazakhstan had major CRC-related mortality changes in 2013 for both men and women. The possible speculated high gain with screening program could be related to no specified upper limit for screening leading to diagnosis of more polyps/CRC in the elderly with high morbidity and mortality risk. It also has the ease of guideline implementation. A recent abstract from 2019, showed for the period 2013 - 2018, the CRC screening was performed in 5,133,602 subjects. The average FIT positivity rate was 1.23% (62,971) and colonoscopy rate was 73.3% leading to diagnosis of CRC in 2,480 patients and 0.07% incidence in high-incidence areas. High colonoscopy rate in FIT-positive patients is a good indicator of appropriate referral system and infrastructure [36]. A similar study in western Kazakhstan concluded that overall CRC incidence increased 1.5-fold through the first decade of the screening implementation and is expected to increase further due to screening program uptake. The differential increase in the diagnosis of stage I CRC vs. advanced stages at diagnosis by 2018, as well as the trend of decreased rates among urban population provides some affirmation to the efficiency of the screening program in the long run [37].

7. Latvia: CRC ranks first in cancer incidence, though the mortality-to-incidence ratio is lowest at 0.39. Latvia has FIT-based nationwide opportunistic yearly CRC screening for people aged 50 to 74 years. The program was started in 2009 and reporting started in 2014. The mortality trends have not shown a sustained decrease. Some possible reasons may be, decreased colonoscopy rates amongst FIT-positive subjects [38]. A 2011 study suggested a significant proportion of CRC were being diagnosed in late stages indicating a need to improve early diagnostics and population-based screening programs [39]. This barrier improved as suggested by data from the latter part of the decade. The participation rate for CRC screening in Latvia was 10.9% for the target population [40]. The uptake of screening was 31.2% for the gFOBT and 44.7% for FIT. In another similar study, FOBT was completed by 4,899 subjects with a response rate of 44.7% [41]. The uptake in the gFOBT group increased with mailing of an advance notification letter with information about FOBT (7.7%, P<0.0001). Additionally, 30.9% returned tests were received after the reminder letter [42]. Average screen positivity leading to colonoscopy indication was 4.1% [43]. Only 150 adequately performed colonoscopies were needed to detect 27 cases with advanced lesions in the target population (cancer or advanced adenoma) [41].

8. Lithuania: Lithuania has a population-based nationwide screening with CRC for people 50-74 years every two years since 2009. The invitations are sent through Primary Health Center. CRC mortality trends have decreased and are significant for males as compared to females. CRC incidence in adults <50 years has also declined in Lithuania [11]. Another study reviewed the screening program in Lithuania, wherein FIT was provided to 271,396 of 890,309 50-74-year-old residents. 251,941 had FIT negative and 19,455 had FIT positive. Only 50% of FIT positive had colonoscopies with 67% showing no pathology. Of 33% of colonoscopies that showed pathology, 3.9% were high-grade neoplasia and 3.1 % cancer. The rate of CRC detected by the screening program was 0.2% [44]. Another similar study found the average time interval from positive stool test result to diagnosis of polyps or CRC was 55 days [45]. It could be inferred that the CRC screening program is effective and could be further improved by increasing invitation coverage and rate of timely referral for colonoscopy after positive FIT [44,45]. According to a European Union 2017 report, the screening coverage with FIT is 57.5% for women and 47% for men. Further screening program also had 100% completeness of data related to screening test and colonoscopy assessment and attendance. However, data was not complete about histological results [43].

9. Russia: Russia has two different regional CRC screenings. Population-based screening in the St. Petersburg region including 18 town districts and non-population-based regional screening in Kazan and Tatarstan republic for people of 48-75 years with FOBT. Though CRC mortality is improving in Russia, it had not reached statistical significance [46]. Similarly, disparities are seen in different regions of Russia. A prospective, multicenter study involving 14 endoscopy centers, selected by the Russian Society of Endoscopy from different regions of the country had good adenoma detection and cecal intubation rate. At multivariate analysis, age, male sex, presence of alarm symptoms, split preparation, cecal intubation rate and withdrawal time measurement were predictors of a higher adenoma detection rate [47]. The surgical outcome of CRC was optimal especially amongst elderly with a 30-day mortality of 4.9%. Cumulative five-year survival was 67.3% and cancer-specific survival was 77.1% [48]. A CRC screening study of a high-risk population including employees of the petrol plant observed a high yield rate for CRC. Among 885 workers screened, adenoma was detected in 34 and CRC in four individuals [49].

10. Other NCAC (Kyrgyzstan, Moldova, Tajikistan, Turkmenistan, Ukraine, Uzbekistan): No additional data exist other than that mentioned in previous table and breakpoint testing. Amongst these countries, only Ukraine has a regional, population-based CRC screening. No data is available from this screening program. Though some changes in CRC mortality trends are noticed in females. For Kyrgyzstan, 19.2% NCD related deaths are related to cancer, one of the lowest in the region.

Summary of CRC Screening in NCAC

Two studies with population sizes of 480 and 2300 who underwent screening colonoscopy in Azerbaijan showed a number needed to detect adenoma or cancer as 5.3 and 6.5 respectively.

Table 1 gives a brief overview of the current CRC screening in NCAC. CRC screening in NCAC is in the initial stage. Few countries have implemented and have an ongoing high-quality population-based cancer registry. Seven NCAC have CRC screening programs with most utilizing non-invasive methods for screening. Data is lacking about the adequacy of colonoscopy preparations, positive predictive value of FIT, procedure-related complication rates and quality of histopathology.

**Table 1:** Colorectal Cancer Screening in North and Central Asian countries Population (P), Opportunistic (O), Nationwide (N), Voluntary (V), Centralized (C), Regional (R), Not available (NA), guaiac fecal occult blood test (gFOBT) or fecal immunochemical tests (FIT), Population based (PB), Non population based (NPB), Digital rectal exam (DRE)

Country	CRC Screening Available	Year of Launching	Type of Screening	Type of Access: Population (P)/ Opportunistic (O), Nationwide (N), Voluntary (V)	Centralized (C)/ Regional (R) Reporting	Data on screening available	Age group (years)	Frequency of screening	Phase of screening	Participation rate
Armenia	Pilot project planned	NA	NA	NA	NA	No	NA	NA	NA	NA
Azerbaijan	No	NA	NA	NA	NA	No	NA	NA	NA	NA
Belarus	No	NA	NA	NA	NA	No	NA	NA	NA	NA
Estonia [33.34]	Yes	2016	FIT	P, N	R	No	60-69	Biennial	Pilot Study	
Georgia [35]	Yes	2006	FOBT	O, N	Tblisi: C Outside Tbilisi: R	Yes	50-69	Biennial		53% in Tblisi, 84% outside Tblisi
Kazakhstan [36,37]	Yes	2011	FOBT	V	R	No	50 and older	Biennial		
Kyrgyzstan	No	NA	NA	NA	NA	No	NA	NA	NA	NA
Latvia [40-43]	Yes	2009, reporting started 2014	FIT	O, N	R	No	50-74	Yearly		
Lithuania [44,45]	Yes	2009	FIT	P, N Invitation sent through PHC	R	No	50-74	Biennial		
Moldova	No	NA	NA	NA	NA	No	NA	NA	NA	NA
Russia [46]	Yes	PB started 2015 NPB started 2010	FOBT (PB) FOBT, DRE questionnaire (NPB)	P in regional St. Petersburg, 18 town districts. NPB regional in Kazan, Tatarstan Republic	R	No	48-75	NA	Pilot Study	
Tajikistan	No	NA	NA	NA	NA	No	NA	NA	NA	NA
Turkmenistan	No	NA	NA	NA	NA	No	NA	NA	NA	NA
Ukraine [46]	Yes	2002-2006	NA	P, N	R	Yes	NA	NA	NA	NA
Uzbekistan	No	NA	NA	NA	NA	No	NA	NA	NA	NA

Table 2 summarizes similar data obtained in other countries which has a wide variation ranging from 2070 FIT tests needed to detect one cancer or adenoma in Kazakhstan to four colonoscopies in Belarus. This table depicts the high sensitivity of diagnosing colon cancer or adenoma using colonoscopy vs other tests although both tests have advantages and disadvantages.

**Table 2:** Colorectal Diagnosis Related Studies in North and Central Asian Countries gFOBT guaiac fecal occult blood test,  FIT fecal immunochemical tests, NA Not applicable

Screening/Research FIT/FOBT/Colonoscopy	Number of participants	Number of completed FIT/FOBT	FIT positivity rate	Number of colonoscopies completed	Total number of adenomas detected	Total number of cancers detected	Adenoma detection rate	Cancer detection rate (number of cancers detected per 100 colonoscopy)
Research/colonoscopy Azerbaijan [28]	480	NA	NA	480	56	34	11.6%	7.1% (symptomatic patients)
Research/colonoscopy Azerbaijan [29]	2300	NA	NA	2300	249	111	10.8%	4.8% (symptomatic patients)
Research /FIT and colonoscopy study Belarus [31]	332	332	32 (9.6%)	332	137 adenoma, 270 polyp	1	41.3%	0.3% (asymptomatic patients)
Screening/ FIT Georgia [35]	1368	1368	54 (4%)	54	Not reported	Not reported	Not reported	Not reported
Screening/FIT Kazakhstan [36]	51,33,602	51,33,602	62,971 (1.23%)	46,158 (73.3%)	NA	2480 (0.05%of screened)	Not reported	5.4% (asymptomatic patients)
Screening/FIT Lithuania [44]	271,396	271,396	19,455 (7.2%)	12,864 (66.1%)	Not reported	Not reported	Not reported	0.2% (asymptomatic patients)
Screening /FIT Russia [49]	885	849	237 (27.9%)	107 (45.1%)	34	4 (0.5% of screened)	31.8%	3.7% (asymptomatic patients)

Discussion

The results suggest that though some countries like Georgia and Latvia have an established screening program, it has not affected their mortality rates. On the other hand, countries like Kazakhstan and Lithuania with screening programs have noticed a positive change in mortality trends. Some factors influencing the screening effectiveness are proper follow-up and referral system, established infrastructure for cancer care and treatment. Additionally, CRC-related screening and treatment cost would be significant modulator of effective screening program in this region.

Among all cancers, CRC ranks first in the incidence rate in Belarus, Latvia, Moldova, Lithuania and Ukraine. Age-standardized incidence rate among men is highest in Moldova (19.6) and 16.8 for women in Latvia. The incidence and the age-standardized mortality rate are higher than other HICs. The countries with very high HDI had high age-standardized incidence rates (Figure 2).

Estonia, Georgia, Kazakhstan, Latvia, Lithuania, Ukraine and Russia have CRC screening programs. Wide variation exists in number needed to detect adenoma or cancer. Further suggesting there is a high sensitivity of diagnosing a colon cancer/adenoma using colonoscopy in this region as compared to FIT or FOBT (Table 2). The adenoma detection rate (ADR) and polyp detection rate (PDR) are low to optimal in this region and cancer detection rates are comparable to other HIC. In a symptomatic cohort from Azerbaijan adenoma detection rate was about 11.6% and cancer detection was 7.1% [28]. The American College of Gastroenterology (ACG) Task Force recommends adenoma detection rates of 30% for men and 20% for women in an average risk individual as a minimum quality measure [50]. The ADR and PDR are additionally higher in symptomatic colonoscopies as compared to screening colonoscopies; ADR rate being 50% as compared to 33% and PDR being 68% vs 51% respectively [51]. Since the prevalence of colonoscopy for symptomatic indications is higher in LMIC, it may be more reasonable to have ADR or PDR per 100 symptom-related colonoscopies as colonoscopy quality indicators for this region.

CRC detection rate is 0.05% for screening in Kazakhstan and 0.2% for screening in Lithuania. These numbers are comparable to screening in HIC and other LMIC with CRC detection rates varying from 0.1 to 0.4% [52,53]. Additional risk factors prevalent in the region increasing the CRC trend includes male liquidators from the Gomel region in Belarus and petrol plant workers in Russia wherein the CRC detection rate was 0.45% [32,49].

All NCAC have a cancer registry, with some having a high-quality registry. However, the format used and the information collected have wide variability among these countries making a comparison of CRC morbidity and mortality rates difficult. For example, Azerbaijan has pathology, population and colonoscopy-based registry [54,55]. Cancer registries should be encouraged to follow a uniform pattern for reporting and case ascertainment according to international guidelines. Studies suggest if the cancer registry is not linked to the death registry then CRC-related deaths are mostly missed in the elderly population [56]. Cancer registries could also help us assess lead time bias. Major variations exist between high-income and low-income countries in the proportion of their populations covered by cancer registries [57].

CRC-related infrastructure including access to surgery, colonoscopy, post-surgical care/outcomes and availability of dedicated cancer centers/physicians are some of the factors that differ among the countries contributing to inter-country survival differences [58]. Thus, strengthening of infrastructure is an important component of CRC screening programs [59]. Nonadherence to guidelines, decreased laboratory quality, delays to subsequent investigation and treatment also affects screening program in this region [34]. Hence, ongoing monitoring for the burden of cancer, societal attitudes towards cancer prevention, cancer health literacy, a referral system for abnormal tests, improving inequitable treatment and access to medicine are vital components of a cancer control program [60,61].

Limitations

Because of scarce data, quantitative assessment for effectiveness of each screening program was not done. Further, abstracts without full articles were also included. The data points obtained from public health agencies have limitations associated with the sources they were derived from. There may be possible data discrepancies since the quality of data collection varies between the NCAC. Further, the website from which the online breakpoint testing was derived has been modified (http://dep.iarc.fr/WHOdb/WHOdb.htm), to improve the graphical presentations of the cancer incidence over time, though it does not have the optional testing for breakpoint to inform about the statistically significant changes in mortality rates. It does have the option to check for annual percentage change with 95% confidence interval that are available for the colorectal cancer mortality for some of the NCAC countries including Estonia, Latvia, Lithuania and Kyrgyzstan. Because of paucity of data, we also included studies that were performed for diagnostic purposes in symptomatic patients for CRC diagnosis. The estimates provided are prior to the impact of coronavirus disease 2019 (COVID-19) as they are based on extrapolations of cancer data collected in earlier years before the pandemic and hence may have changed recently.

## Conclusions

High HDI NCAC countries have higher incidence of CRC and mortality. Screening programs are not available in all the countries except in some very high to high HDI countries and the most common methods are FIT and FOBT. Studies with colonoscopy indicated for symptoms have shown good adenoma detection rates. Data is being reported regionally and most countries don’t have organized data collection registries making population-based studies difficult. As CRC is the leading cause of cancer in many NCAC, governments should focus on screening programs to increase the early detection of CRC. Maintaining international guidelines-based standard registries should be a priority to facilitate population-based studies to assess the actual disease burden.

Considerable literature shows the efficacy and cost-effectiveness of CRC screening tests in resource-limited countries. However, very limited information is available on the actual cost and logistics of implementing a CRC screening program. Further research is needed to evaluate cancer screening implementation and dissemination in North and Central Asian countries.
